# Pharmacokinetics, Excretion, and Metabolite Profiling of Leonurine in Rats: Evidence for Extensive Phase II Conjugations

**DOI:** 10.3390/molecules31122002

**Published:** 2026-06-08

**Authors:** Xu Liu, Jing Hu, Yang Chen, Bin Shi, Zhanpeng Shang, Yan Liang

**Affiliations:** 1 Department of Pharmacy, Medical Supplies Center of PLA General Hospital, Beijing 100853, China; 2 Senior Department of Traditional Chinese Medicine, Chinese PLA Hospital, Beijing 100853, China; 3 Department of Organ Transplantation, The Third Medical Center of PLA General Hospital, Beijing 100039, China; 4 School of Pharmaceutical Sciences, Peking University, Beijing 100083, China

**Keywords:** leonurine, pharmacokinetics, excretion, metabolite profiling, glucuronidation, sulfation

## Abstract

Leonurine, a bioactive alkaloid from *Leonurus japonicus*, has attracted considerable pharmacological interest, yet its in vivo disposition remains insufficiently defined. In the present study, the pharmacokinetics, excretion, and metabolic profile of leonurine were systematically investigated in rats after intravenous (IV), oral (PO), and intraperitoneal (IP) administration. A validated LC–MS/MS method was used to quantify leonurine in plasma, urine, and feces, and high-resolution MS was applied for metabolite profiling. Following IV administration, leonurine exhibited rapid systemic disposition, with a half-life of 2.48 h and a clearance of 152 mL/min/kg. Oral exposure was negligible, with an absolute bioavailability of 0.14%, whereas IP administration produced markedly higher systemic exposure (66.6%). Recovery of unchanged leonurine in urine and feces remained low across all dosing routes, with total excretory recovery below 6% of doses. The results indicated that metabolic conversion, rather than parent drug excretion, was the dominant elimination pathway. A total of 30 leonurine-related components were characterized in vivo, including 24 previously unreported metabolites. The metabolic profile was dominated by phase II conjugation, comprising 12 glucuronidated and 12 sulfated metabolites, together with hydrolysis, methylation/demethylation, and other transformation products. Notably, ester bond cleavage was identified as one of the major primary biotransformation routes, and several glucuronide and sulfate conjugates were also formed on hydrolysis-derived fragments. These findings provide a more comprehensive view of leonurine disposition in rats and offer a mechanistic basis for its rapid clearance and limited systemic availability after oral administration.

## 1. Introduction

Leonurine is a distinctive guanidino alkaloid predominantly found in plants of the *Leonurus* genus [1], especially in *Leonurus japonicus* (*Herba Leonuri*), a traditional medicinal herb widely used for gynecological disorders [2,3]. *Herba Leonuri* contains multiple classes of chemical constituents, including alkaloids, flavonoids, diterpenes, iridoid glycosides, phenylpropanoids, sterols, peptides, and phenolic glycosides, among which alkaloids are considered important bioactive components [4]. As one of the representative alkaloids isolated from *Herba Leonuri*, leonurine has been isolated from *Herba Leonuri* using various extraction and purification strategies, including acidic methanol extraction, ethanol or aqueous extraction, chromatographic purification, high-performance liquid chromatography, high-speed countercurrent chromatography, and acidic ionic liquid-assisted ultrasonic extraction [4]. Recently, leonurine has attracted increasing attention because of its broad spectrum of pharmacological activities, including uterotonic [5], anti-inflammatory [6], antioxidant [7], cardioprotective [8,9], and neuroprotective effects [10]. Recent reviews have further summarized the natural origin, extraction and purification, chemical synthesis, structural modification, and pharmacological properties of leonurine, highlighting its potential as a natural-product-derived lead compound for cardiovascular, neurological, and other chronic diseases [4,11].

Despite its favorable pharmacological profile, the in vivo disposition of leonurine remains insufficiently characterized [12,13,14,15]. Available evidence suggests that leonurine exhibits unfavorable pharmacokinetic behavior, particularly low oral bioavailability [15]. This limitation may substantially compromise its systemic exposure and translational potential. To date, metabolism studies of leonurine have been relatively limited [12,13,15]. In an early rat study after oral administration, three metabolites were reported, including one glucuronide, one sulfate, and one *O*-demethylated metabolite, and the major glucuronide was structurally confirmed as leonurine-10-*O*-β-D-glucuronide by NMR [15]. Subsequent in vitro investigations in human liver and intestinal microsomes demonstrated that *O*-glucuronidation is a predominant metabolic pathway of leonurine, with UGT1A1 identified as the principal enzyme responsible for glucuronidation, while demethylation was also observed as a primary transformation [13]. Although these studies established conjugative metabolism as an important clearance route, they did not provide a systematic picture of leonurine disposition in vivo, particularly with respect to the relationship among systemic pharmacokinetics, excretion of unchanged parent drug, and metabolic transformation. Such information is essential not only for clarifying the major elimination pathways of leonurine, but also for guiding future studies on structural optimization and translational development.

Therefore, the present study was designed to systematically investigate the pharmacokinetics, excretion, and metabolic profile of leonurine in rats. Specifically, leonurine disposition was evaluated after intravenous (IV), oral (PO), and intraperitoneal (IP) administration, the recovery of unchanged leonurine in urine and feces was also quantified, and high-resolution MS/MS-based metabolite identification was performed in plasma, urine, and feces. By integrating these three aspects, this study aimed to provide a more complete understanding of the in vivo fate of leonurine and to elucidate the major pathways contributing to its limited oral exposure and overall elimination.

## 2. Results

### 2.1. Method Validation

**Figure 1:**
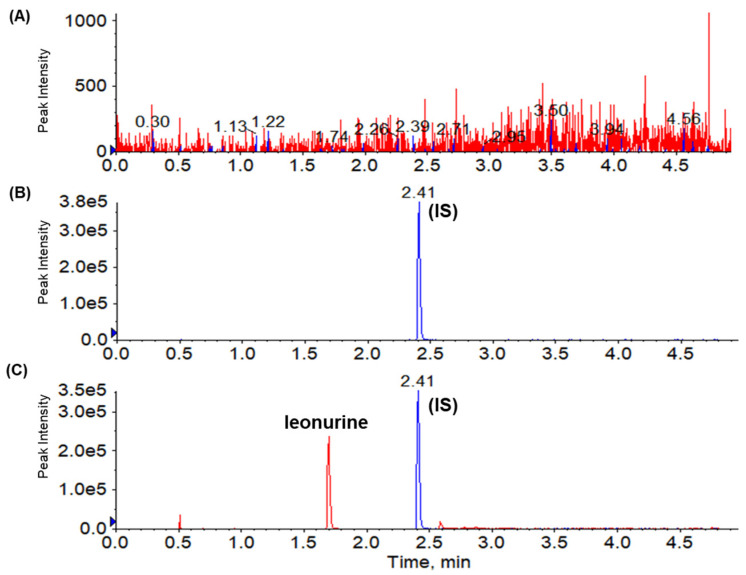
Representative MRM chromatograms of a blank biological sample (**A**), a blank biological sample spiked with the internal standard (IS) (**B**), and a blank biological sample spiked with leonurine (10 ng/mL) and IS (**C**).

Representative multiple reaction monitoring (MRM) chromatograms of blank plasma, a blank sample spiked with the internal standard (IS, terfenadine), and a blank sample spiked with both leonurine and IS are shown in Figure 1. No endogenous interference was detected in the monitored MRM channels, and leonurine was well resolved from the IS.

Calibration curves for leonurine in plasma, urine, and feces were linear over the range of 0.5–1000 ng/mL. The regression equations were *y* = 0.0146*x* + 0.00209 (*r* = 0.9931) for plasma, *y* = 0.0315*x* + 0.0102 (*r* = 0.9963) for urine, and *y* = 0.0324*x* + 0.0156 (*r* = 0.9977) for feces. The lower limit of quantification (LLOQ) met the acceptance criteria for bioanalytical quantification, with accuracy within ±15% relative error (RE), whereas the remaining calibration levels were within ±10% RE.

As summarized in Tables S1 and S2, the method showed acceptable intra-day and inter-day precision and accuracy. Intra-day RSD values were 7.03%, 4.25%, and 4.20%, and inter-day RSD values were 4.25%, 4.17%, and 3.28% at the low, medium, and high quality control (QC) levels, respectively. Mean accuracies were 97.3%, 87.1%, and 92.1%. Extraction recovery ranged from 88.1% to 98.0%, and matrix effects ranged from 93.1% to 101%, indicating efficient extraction and negligible matrix interference. Leonurine was stable in rat plasma for at least 4 h at 24 °C, 24 h at 4 °C in the autosampler, and 14 days at −80 °C.

These results demonstrated that the established LC–MS/MS method was robust, accurate, and precise, and was therefore suitable for quantification of leonurine in rat biological matrices for pharmacokinetic (PK) and excretion studies. Terfenadine was selected as the IS for leonurine quantification because it provided a stable response, appropriate chromatographic behavior, and no endogenous interference in blank rat plasma. Its suitability was further supported by the above acceptable recovery, matrix effect, accuracy, and precision obtained during method validation.

### 2.2. Pharmacokinetics and Excretion Studies

**Figure 2:**
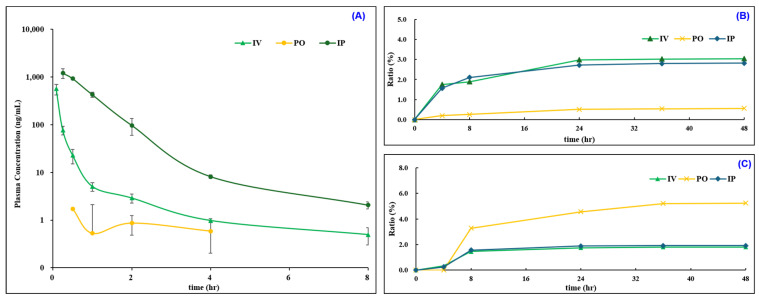
Mean plasma concentration-time profiles of leonurine (**A**) and cumulative excretion of unchanged leonurine in urine (**B**) and feces (**C**) after IV, PO, and IP administration.

The validated LC-MS/MS method was applied to investigate the PK and excretion of leonurine in rats following IV, PO, and IP administration. PK parameters derived from noncompartmental analysis are summarized in Table 1, and the mean plasma concentration–time profiles (*n* = 5) are illustrated in Figure 2A.

Following IV administration, leonurine concentrations declined rapidly in plasma. The key PK parameters were elimination half-life (T_1/2_) = 2.48 ± 0.37 h, mean resistance time (MRT) = 0.41 ± 0.12 h, area under the concentration–time curve from zero to the last measurable time point (AUC_0–t_) = 170 ± 41.2 h·ng/mL, apparent volume of distribution (V_d_) = 3.85 ± 1.76 L/kg, initial plasma concentration (C_0_) = 1513 ± 428 ng/mL, and total body clearance (CL) = 152 ± 38.8 mL/min/kg. After PO administration, systemic exposure to leonurine was extremely limited, with maximum plasma concentration (C_max_) = 1.85 ± 1.45 ng/mL and AUC_0–t_ = 3.14 ± 1.82 h·ng/mL, corresponding to an absolute bioavailability (F) of only 0.14 ± 0.08%. By comparison, IP administration produced markedly greater exposure than PO dosing, with C_max_ = 1210 ± 263 ng/mL, AUC_0–t_ = 1138 ± 130 h·ng/mL, and F = 66.6 ± 7.58%. These data indicated that leonurine is rapidly cleared from the systemic circulation and exhibits negligible oral bioavailability in rats. The pronounced difference between PO and IP exposure further suggested that the poor oral exposure cannot be explained solely by rapid systemic clearance, but is also likely associated with presystemic loss, including limited gastrointestinal absorption and/or intestinal/hepatic first-pass metabolism [13,15,16]. A previous LC-MS/MS study reported the pharmacokinetic behavior of leonurine in rat plasma after oral administration of Herba Leonuri extract, in which leonurine showed relatively low systemic exposure with a C_max_ of 43.3 ± 8.2 ng/mL and an AUC_0–t_ of 75.4 ± 20.1 ng·h/mL [17]. This finding is generally consistent with the present observation that leonurine exhibited limited oral exposure and rapid in vivo disposition [18].

This interpretation was reinforced by the excretion profiles of unchanged leonurine in urine and feces. Over 48 h after dosing, cumulative urinary recovery of the parent compound remained low for all administration routes, reaching only 3.04%, 0.56%, and 2.81% of dose after IV, PO, and IP administration, respectively. Fecal recovery of unchanged leonurine was likewise limited after IV and IP administration, accounting for 1.80% and 1.92% of dose, respectively, whereas a relatively higher proportion was recovered in feces after PO administration (5.23%). Consequently, the total excretory recovery of unchanged leonurine (urine plus feces) remained below 6% for all three dosing routes, amounting to 4.84%, 5.79%, and 4.73% after IV, PO, and IP administration, respectively. After IV and IP dosing, most of the parent drug recovered in excreta appeared within the first 24 h, followed by only minimal additional recovery, indicating that elimination of leonurine as the intact parent compound makes only a minor contribution to overall clearance. Combined with the extremely low systemic exposure, this finding suggested that poor gastrointestinal absorption (F_a_) and/or intestinal first-pass metabolism (F_g_) might be major determinants of the negligible oral bioavailability [13].

### 2.3. Workflow to Characterize Leonurine Metabolites in Rats

#### 2.3.1. High Resolution MS/MS Data-Acquisition

In contrast to previous leonurine metabolite studies [13,15], which were mainly conducted using a single ionization mode, the MS acquisition strategy in the present work was specifically optimized according to the structural characteristics of leonurine. Because leonurine contains a potentially labile ester linkage, cleavage of this bond was expected to generate two chemically distinct metabolite subclasses, namely carboxylic acid-containing fragments and guanidine-containing aliphatic alcohol fragments. Owing to their different ionization behaviors, the former were expected to be detected more efficiently in the negative ion mode, whereas the latter were anticipated to show higher responses in the positive ion mode. Therefore, data acquisition was performed in both ionization modes to improve coverage of structurally diverse metabolites.

To further enhance MS/MS acquisition efficiency, dynamic exclusion was introduced based on chromatographic peak width and signal characteristics observed in the control samples. The exclusion parameters were set at 2 s for apex trigger and 3 s for exclusion duration [18,19]. This setup reduced redundant fragmentation of repeatedly selected precursor ions and increased the diversity of acquired product-ion spectra. As a result, the final acquisition workflow provided broad metabolite coverage together with high-quality MS/MS data, supporting the subsequent structural characterization of leonurine-related metabolites.

#### 2.3.2. MS/MS Fragmentation Behaviors of Leonurine

**Figure 3:**
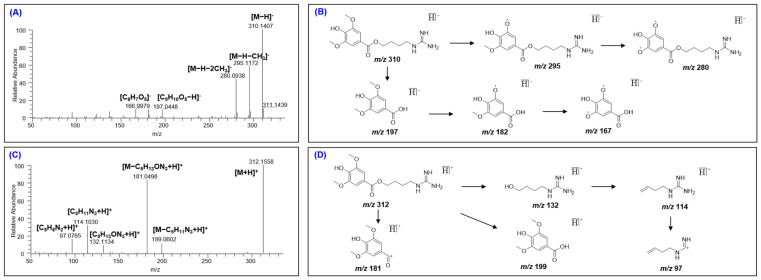
The MS/MS spectra and proposed fragmentation pathways of leonurine in the negative ion mode (**A**,**B**) and positive ion mode (**C**,**D**).

To support subsequent metabolite assignment, the MS/MS fragmentation behavior of leonurine was first characterized in both negative and positive ionization modes, which provided complementary structural information for the two major substructures of the molecule. In the negative ion mode, leonurine produced a deprotonated molecular ion at *m*/*z* 310.1407 ([M-H]^−^, error 1.06 ppm, Figure 3A,B). The fragmentation pattern was dominated by successive demethylation, giving rise to product ions at *m*/*z* 295.1172 and *m*/*z* 280.0938, corresponding to the loss of one and two methyl groups, respectively. In addition to these neutral losses, cleavage of the ester bond generated a prominent aromatic carboxylate-derived ion at *m*/*z* 197.0448, which was further converted to *m*/*z* 182.0207 and *m*/*z* 166.9979 through sequential demethylation.

In the positive ion mode, leonurine generated a protonated molecular ion at *m*/*z* 312.1558 ([M+H]^+^, error 1.12 ppm, Figure 3C,D). In contrast to the negative mode, fragmentation under positive ionization was mainly characterized by ester bond cleavage together with decomposition of the guanidine-containing side chain. The major product ion at *m*/*z* 181.0498 was assigned to cleavage of the ester linkage with loss of the guanidinoalkyl moiety, whereas *m*/*z* 199.0602 represented the complementary aromatic ester-derived fragment. The guanidinoalkyl portion further generated a series of diagnostic ions at *m*/*z* 132.1134, *m*/*z* 114.1030, and *m*/*z* 97.0765, reflecting stepwise fragmentation of the side chain.

#### 2.3.3. MS/MS Fragmentation Behaviors of Known Metabolites

Glucuronidation and sulfation have previously been recognized as important metabolic pathways of leonurine in vivo [16]. Earlier studies identified leonurine glucuronide and leonurine sulfate in rats, and leonurine *O*-glucuronide has been shown to be the predominant conjugate in human liver and intestinal microsomes [13]. In the present study, both glucuronidated and sulfated metabolites exhibited highly characteristic neutral-loss (NL) behavior, reflecting the lability of the corresponding conjugated moieties.

**Figure 4:**
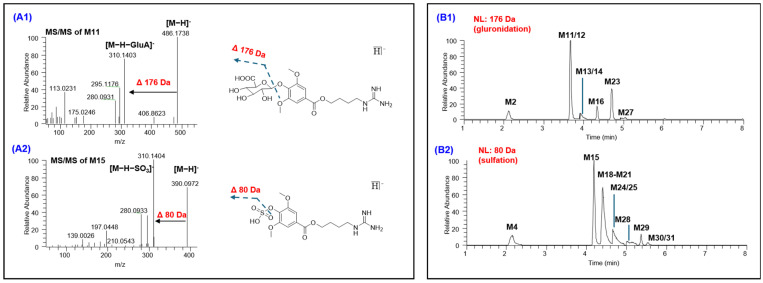
The MS/MS fragmentation behaviors of representative glucuronidated (M11, (**A1**)) and sulfated (M15, (**A2**)) metabolites of leonurine, and screening results based on characteristic neutral-loss fragments in rat plasma after IP administration (**B1**,**B2**).

As a representative glucuronidated metabolite, M11 showed [M-H]^−^ ion at *m*/*z* 486.1738 (Figure 4(A1)). Its MS/MS spectrum was dominated by the product ion at *m*/*z* 310.1403, generated by a diagnostic NL of 176 Da, corresponding to cleavage of the glucuronic acid moiety and regeneration of the leonurine aglycone. Further fragmentation of the aglycone ion produced *m*/*z* 295.1176 and *m*/*z* 280.0931, consistent with the established fragmentation pattern of leonurine. Similarly, the representative sulfate M15 displayed [M-H]^−^ ion at *m*/*z* 390.0972 and generated a dominant ion at *m*/*z* 310.1401 after a diagnostic NL of 80 Da (Figure 4(A2)). Additional fragment ions at *m*/*z* 280.0933, *m*/*z* 210.0543, *m*/*z* 197.0448, and *m*/*z* 139.0026 were also observed, reflecting subsequent fragmentation of the desulfated product ion. These results indicate that NL 176 Da and NL 80 Da serve as highly diagnostic features for leonurine glucuronides and sulfates, respectively.

Based on these fragmentation characteristics, targeted screening of plasma samples collected after IP administration enabled efficient detection of phase II metabolites. Using NL of 176 Da as the diagnostic criterion, 8 glucuronidated metabolites were initially screened (Figure 4(B1)), whereas 12 sulfated metabolites were detected using NL of 80 Da (Figure 4(B2)) as the corresponding screening criterion. These results demonstrated that neutral-loss-guided screening is a practical and effective strategy for systematic discovery of leonurine phase II metabolites in complex biological matrices.

### 2.4. Identification of Leonurine Metabolites

A systematic metabolite profiling of leonurine was performed in rat plasma, urine, and feces following IV, PO, and IP administration using high-resolution MS and MS/MS operated in both negative and positive ionization modes (Table 2). Metabolite annotation was based on an integrated evaluation of accurate MS and MS/MS, elemental composition, characteristic neutral-loss behavior of conjugates, and fragment-ion mapping relative to the parent compound and key hydrolysis-derived intermediates. In total, 30 leonurine-related components, including the parent drug (M25), were characterized in vivo.

#### 2.4.1. Hydrolysis and Other Primary Transformations

**Figure 5:**
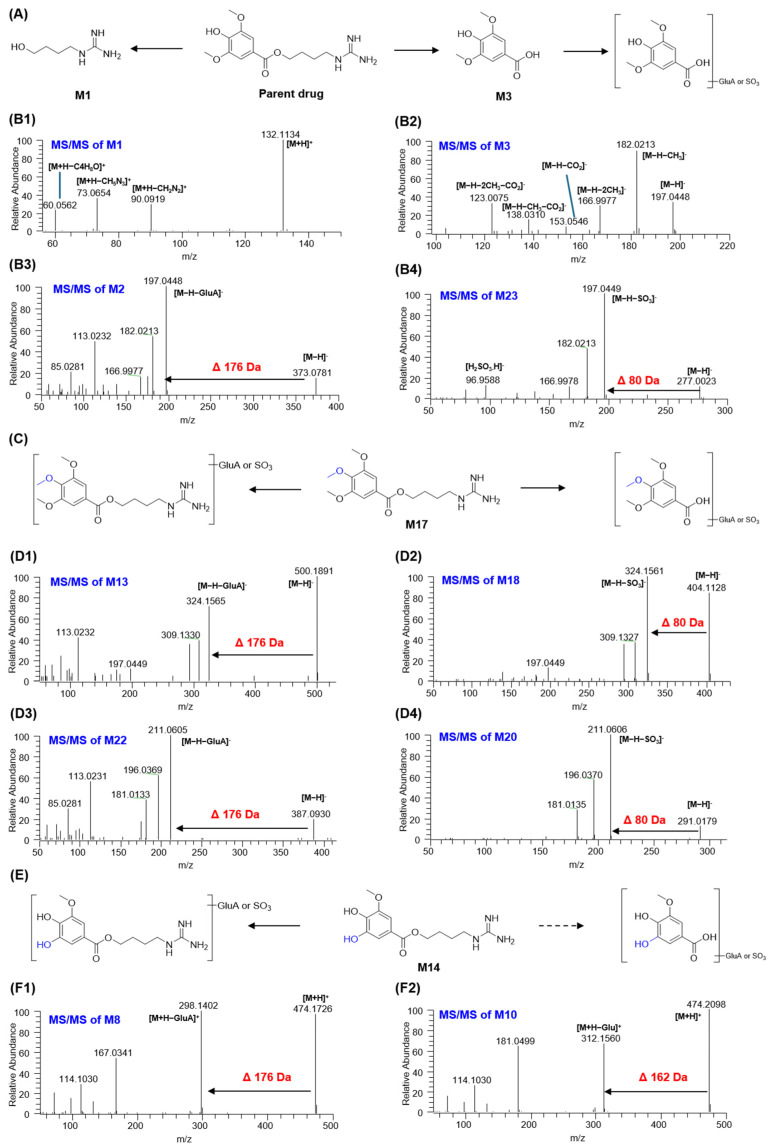
Proposed metabolic pathways and MS/MS spectra of representative leonurine metabolites. (**A**) hydrolysis and corresponding phase II conjugation pathways of leonurine; (**B**) MS/MS spectra of M1 (4-guanidino-1-butanol, (**B1**)), M3 (syringic acid, (**B2**)), M4 (**B3**), and M23 (**B4**); (**C**) hydrolysis of methylated leonurine and the corresponding phase II conjugation pathways; (**D**) MS/MS spectra of M13 (**D1**), M18 (**D2**), M20 (**D3**), and M22 (**D4**); (**E**) Metabolic pathways of demethylated leonurine; (**F**) MS/MS spectra of M8 (**F1**) and M10 (**F2**).

Ester bond cleavage was identified, for the first time, as one of the major primary metabolic pathways of leonurine. This reaction generated two principal hydrolysis-derived products, namely a guanidinoalkyl alcohol fragment (M1) and an aromatic acid fragment (M3) (Figure 5A). M1 was detected in the positive ion mode with the [M+H]^+^ ion at *m*/*z* 132.1134. Its structure was supported by a series of diagnostic product ions at *m*/*z* 90.0919 ([M+H-CH_2_N_2_]^+^), *m*/*z* 73.0654 ([M+H-CH_5_N_3_]^+^), and *m*/*z* 60.0562 ([M+H-C_4_H_8_O]^+^), consistent with stepwise fragmentation of the guanidinoalkyl alcohol moiety released after ester cleavage (Figure 5(B1)). M3 was detected in the negative ion mode at *m*/*z* 197.0448 ([M-H]^−^) and generated characteristic fragment ions at *m*/*z* 182.0213 ([M-H-CH_3_]^−^), *m*/*z* 166.9977 ([M-H-2CH_3_]^−^), together with a decarboxylation ion at *m*/*z* 123.0075 ([M-H-CO_2_]^−^) (Figure 5(B2)). Comparison with an authentic reference standard confirmed that M3 was syringic acid. Besides hydrolysis, other primary transformations were also observed. M14 was assigned as a demethylated metabolite ([M+H]^+^ at *m*/*z* 298.1406), whereas M17 was identified as a methylated metabolite ([M-H]^−^ at *m*/*z* 324.1563). A leonurine glucosylated metabolite, M10, was observed at *m*/*z* 474.2098 ([M+H]^+^) and generated *m*/*z* 312.1560 after NL of 162 Da (Figure 5(F2)).

#### 2.4.2. Characterization of Glucuronide Conjugates

A total of 12 glucuronidated metabolites (M2, M5-M9, M11-M13, M16, M22, and M26) were identified). These metabolites were characterized by the diagnostic NL of 176 Da, corresponding to cleavage of the glucuronic acid moiety.

##### Intact-Scaffold Glucuronides

Among the 12 glucuronides, 8 metabolites (M5-M9, M11, M13, and M16) were assigned as conjugates formed on the intact leonurine scaffold or its methylated/demethylated derivatives. M11 and M16 were two representative isomeric glucuronides, both detected as [M-H]^−^ ions at *m*/*z* 486.17. In their MS/MS spectra, the dominant product ions at *m*/*z* 310.14 were generated *via* NL of 176 Da. The resulting aglycone ion further yielded *m*/*z* 295.12 and *m*/*z* 280.09, consistent with the characteristic negative-ion fragmentation pattern of leonurine. This direct “conjugate to parent drug ion” relationship strongly supported their assignment as leonurine glucuronides. Among them, M11 has been previously reported and was unequivocally identified as leonurine-10-*O*-β-D-glucuronide by NMR [15]. By contrast, M16, which exhibited the same elemental composition and aglycone-mapping fragmentation pattern but exhibited a distinct chromatographic behavior, was tentatively assigned as an *N*-glucuronide isomer.

M13, assigned as a methylation and glucuronidation metabolite, was detected as [M-H]^−^ at *m*/*z* 500.1891 and generated *m*/*z* 324.1565 through NL of 176 Da, matching the independently observed methylated aglycone M17 (Figure 5(D1)). Because methylation of leonurine is expected to block the only phenolic hydroxyl group on the aromatic ring, the glucuronidation in M13 was tentatively assigned as *N*-glucuronidation, most plausibly involving the guanidine moiety. In the positive ion mode, M5, M7, M8, and M9 showed similar precursor ions at *m*/*z* 474.17 ([M+H]^+^) and produced *m*/*z* 298.14 *via* NL of 176 Da. Their additional product ions at *m*/*z* 167.03, *m*/*z* 132.11, and *m*/*z* 114.10 were consistent with the MS/MS behavior of the demethylated aglycone M14 (Figure 5(F1)), supporting their assignment as glucuronides of demethylated leonurine. In addition, M6 was assigned as a diglucuronide, detected as [M+H]^+^ at *m*/*z* 650.2053, with sequential NL of 176 Da generating *m*/*z* 474.1730 and *m*/*z* 298.1402, consistent with stepwise cleavage of two glucuronic acid moieties.

##### Hydrolysis-Derived Glucuronides

The remaining 4 glucuronidated metabolites (M2, M12, M22, and M26) were assigned as hydrolysis-derived glucuronides. M2 and M12 showed [M-H]^−^ ions at *m*/*z* 373.08 and generated *m*/*z* 197.04 after NL of 176 Da, corresponding to the hydrolysis-derived aromatic fragment M3. These data indicated that M2 and M12 were glucuronides formed on the aromatic acid scaffold. M22 and M26 showed precursor ions at *m*/*z* 387.09 ([M-H]^−^) and yielded *m*/*z* 211.06 after NL of 176 Da (Figure 5(D3)), consistent with glucuronidation of a methylated hydrolysis-derived aromatic fragment.

#### 2.4.3. Characterization of Sulfated Conjugates

A total of 12 sulfated metabolites were identified (M4, M15, M18-M21, M23, M24, and M27-M30), all exhibiting a characteristic NL of 80 Da corresponding to sulfate cleavage.

##### Intact-Scaffold Sulfates

Among them, 7 metabolites (M15, M18, M19, M21, M24, M28, and M29) were assigned as sulfates formed on the intact leonurine scaffold or its methylated/demethylated derivatives. M15 and M28 showed [M-H]^−^ at *m*/*z* 390.10 and generated *m*/*z* 310.14 through NL of 80 Da, corresponding to the deprotonated parent drug ion, indicating sulfation of leonurine. Based on previous structural characterization, M15 was assigned as an *O*-sulfation metabolite, whereas M28 was tentatively assigned as an *N*-sulfation metabolite [20,21,22].

M18 and M24, assigned as methylation and sulfation metabolites of leonurine, showed [M-H]^−^ ions at *m*/*z* 404.11 and yielded *m*/*z* 324.16 after NL of 80 Da, matching the methylated aglycone M17 (Figure 5(D2)). Because methylation eliminates the only phenolic hydroxyl group available for conventional *O*-sulfation, the sulfation in M18 and M24 was tentatively assigned as *N*-sulfation, most plausibly occurring on the guanidine moiety. Although *N*-sulfation is a recognized pathway for certain amine-containing drugs [23], assignment to guanidinium-containing metabolites should remain provisional.

Similarly, M19 and M21, both assigned as demethylation and sulfation metabolites, showed [M-H]^−^ ions at *m*/*z* 376.08 and generated *m*/*z* 296.12 after NL of 80 Da, consistent with sulfation of a demethylated leonurine-related scaffold. As the sulfation site could not be unequivocally localized by MS/MS, these metabolites were only described as demethylation-sulfation products, with guanidine *N*-sulfation remaining a plausible but tentative interpretation.

##### Hydrolysis-Derived Sulfates

The remaining five sulfated metabolites (M4, M20, M23, M27, and M30) were assigned as hydrolysis-derived sulfate conjugates. M4 and M23 showed [M-H]^−^ ions at *m*/*z* 277.00 and generated the hydrolysis-derived aglycone ion (M3) at *m*/*z* 197.04 *via* NL of 80 Da (Figure 5(B4)). Because M3 contains only one phenolic hydroxyl group in addition to the carboxyl group, the exact sulfation site could not be unambiguously determined. These metabolites were therefore assigned as hydrolysis-derived sulfate conjugates of M3, with the sulfation position left unassigned.

Similarly, M20 and M30 showed [M-H]^−^ ions at *m*/*z* 291.02 and yielded *m*/*z* 211.06 after NL of 80 Da (Figure 5(D4)), followed by fragment ions at *m*/*z* 196.04 and *m*/*z* 181.01, indicating sulfation of a methylated hydrolysis-derived aromatic fragment. Considering the likely lability of sulfate conjugation at the carboxyl group, the methylation in M20 and M30 was tentatively assigned to the carboxyl group, leaving the aromatic hydroxyl group as the most plausible site for sulfation. Carboxyl methylation is biochemically feasible, although it is generally not regarded as a major pathway in mammalian xenobiotic metabolism.

**Figure 6:**
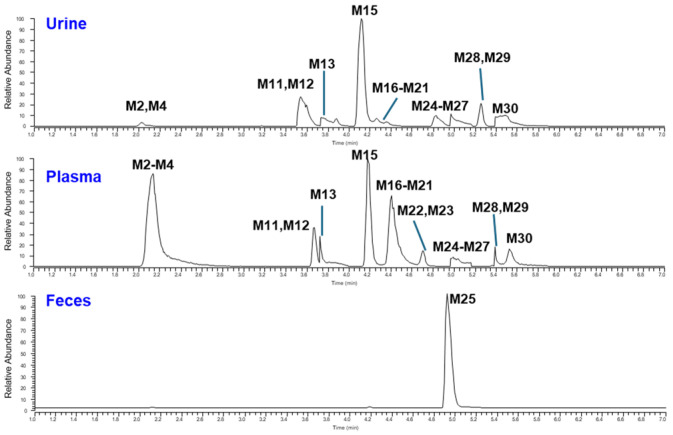
Extracted ion chromatograms of leonurine metabolites detected in rat plasma, urine, and feces after IP administration in the negative ion mode.

For illustration, metabolites identified in plasma, urine, and feces from the IP administration group are shown in Figure 6.

### 2.5. Proposed Metabolic Pathways of Leonurine

**Figure 7:**
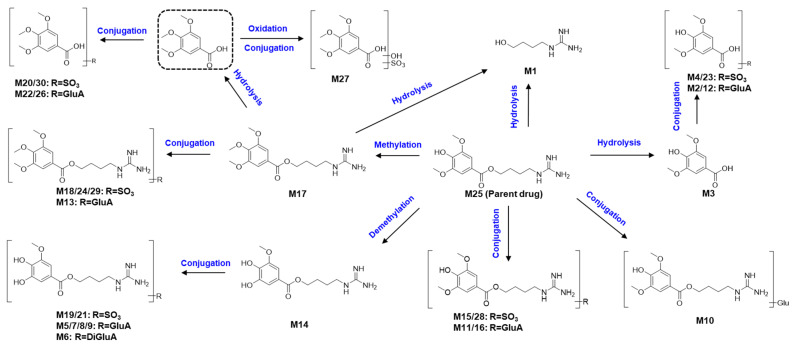
The proposed metabolic pathways of leonurine in rats. (GluA: glucuronidation; SO_3_: sulfation; Glu: glucosylation).

The proposed metabolic pathways of leonurine were summarized in Figure 7 based on the 30 characterized metabolites. Three principal types of biotransformation were revealed. First, leonurine underwent primary structural modifications, among which ester bond cleavage was identified as a major initial event. This transformation generated the two key hydrolysis products, M1 and M3, which subsequently served as important aglycone precursors for downstream metabolism. In addition to hydrolysis, methylation, demethylation, and occasional oxidation were also observed. These modified intermediates also participated in subsequent phase II metabolism. These primary transformations are generally consistent with previous reports describing *O*-demethylation and conjugative metabolism of leonurine in rats and human microsomes.

The second major route was glucuronidation, which occurred in two distinct forms: direct glucuronidation of the intact leonurine scaffold or its methylated/demethylated derivatives, and glucuronidation of hydrolysis-derived aromatic fragments. The third major pathway was sulfation, which was likewise observed both on intact leonurine-related scaffolds and on hydrolysis-derived fragments. In several hydrolysis-derived sulfates, the exact conjugation sites could not be definitively assigned from the available MS/MS data, and some structural annotations therefore remained tentative. In addition, a minor glucosylation pathway was also observed, indicating that leonurine can undergo alternative sugar conjugation in vivo.

These results showed that leonurine metabolism in rats was characterized by extensive phase II conjugation superimposed on a network of primary transformations, with ester bond cleavage representing a particularly important branching step in the overall metabolic scheme.

## 3. Discussions

The present study demonstrated that leonurine underwent rapid systemic disposition and extensive metabolic elimination in rats. Following IV administration, leonurine showed a short elimination half-life and high systemic clearance, whereas PO dosing resulted in negligible systemic exposure and extremely low oral bioavailability. By contrast, IP administration produced markedly higher exposure than PO dosing. These findings indicated that the poor oral exposure of leonurine cannot be attributed solely to rapid systemic clearance, but was more likely associated with first-pass effect, including intestinal and/or hepatic first-pass metabolism. This interpretation was further supported by the excretion results, as the total recovery of unchanged leonurine in urine and feces remained below 6% of dose after IV, PO, and IP administration, indicating that direct excretion of the parent drug contributes only minimally to overall elimination. Collectively, the pharmacokinetic and excretion data consistently point to metabolic conversion, rather than unchanged parent excretion, as the dominant determinant of leonurine disposition in rats.

The metabolite profiling results substantially expanded current knowledge of leonurine biotransformation. Whereas earlier rat studies reported three metabolites, and subsequent human microsomal studies established *O*-glucuronidation as a predominant pathway mainly mediated by UGT1A1, the present study characterized 30 leonurine-related components, including 12 glucuronides, 12 sulfates, and several phase I metabolites. Most importantly, ester bond cleavage was identified as one of the major primary biotransformation routes, generating the guanidinoalkyl alcohol fragment (M1) and the aromatic acid fragment (M3), which subsequently acted as key branching intermediates for downstream metabolism. The identification of both glucuronidated and sulfated conjugates on the intact leonurine scaffold as well as on hydrolysis-derived aromatic fragments supports a stepwise metabolic sequence in which hydrolysis is followed by additional phase I and phase II transformations. This metabolic pattern provided a mechanistic explanation for the very low recovery of unchanged leonurine in excreta and the negligible oral exposure observed after PO administration, and was also consistent with previous in vitro evidence that leonurine undergoes efficient conjugative metabolism in liver and intestinal microsomes [13,15,16].

Another important feature of the current metabolic pathways was the structural diversity of conjugates, which were detected not only on leonurine itself but also on methylated/demethylated and hydrolysis-derived metabolites, indicating that sulfation, together with glucuronidation, represented a major detoxification route for multiple leonurine-related intermediates. At the same time, several positional assignments—particularly for putative *N*-glucuronides, *N*-sulfates, and certain hydrolysis-derived sulfate conjugates—remain tentative and require orthogonal confirmation. Furthermore, because the present study did not include bile duct-cannulated animals, recombinant enzyme phenotyping, or microbiota-oriented models, the relative contributions of hepatic, intestinal, and possible microbial metabolism could not be distinguished. Nevertheless, the current findings provide a coherent explanation for the in vivo disposition of leonurine in rats, showing that its rapid systemic clearance, minimal excretion as unchanged parent drug, and poor oral availability were closely associated with extensive conjugative biotransformation superimposed on a major hydrolytic branching pathway.

The biological relevance of the identified metabolites should also be considered. M1 may be of particular interest because a structurally related guanidino alcohol, 3-guanidinyl propanol, has been reported to enhance salt taste through a TMC4-mediated current [24]. This observation suggests that guanidino-containing metabolites derived from leonurine may not be merely inactive degradation products, but could possess distinct functional properties. The aromatic acid-related metabolites identified in this study may also have biological relevance. Syringic acid, generated through hydrolytic cleavage of the ester linkage of leonurine, is a naturally occurring phenolic acid with reported antioxidant and anti-inflammatory activities [25]. Mechanistically, syringic acid has been described to mitigate oxidative stress by scavenging free radicals, enhancing endogenous antioxidant defenses, and activating the KEAP1/NRF2 pathway, while suppressing inflammatory signaling through downregulation of NF-κB-, TLR4-, HMGB1-, MyD88-, and TRAF6-related pathways [26]. Previous studies and reviews have also associated syringic acid with neuroprotective, cardioprotective, hepatoprotective, and antidiabetic effects [27]. Therefore, the formation of syringic acid may not only represent a clearance pathway of leonurine but may also have potential implications for its overall biological profile. Further studies using authentic metabolite standards, quantitative exposure assessment, and activity-based assays will be necessary to determine whether these metabolites contribute to the therapeutic or biological effects of leonurine.

## 4. Materials and Methods

### 4.1. Chemicals and Reagents

Methanol and acetonitrile (LC-MS grade) were purchased from Fisher Scientific (Waltham, MA, USA). Formic acid (LC-MS grade) was obtained from Sigma-Aldrich (St. Louis, MO, USA). Deionized water was prepared using a Milli-Q purification system (Millipore, Burlington, MA, USA). Leonurine and terfenadine (IS), both with a purity > 98%, were supplied by Macklin Biochemical Technology Co., Ltd. (Shanghai, China). Grace Pure™ C18 low solid-phase extraction cartridges (200 mg/3 mL) were obtained from Grace Davison Discovery Science (Deerfield, IL, USA).

### 4.2. Animals

Fifteen male Sprague-Dawley (SD) rats (body weight: 200 ± 20 g) were obtained from SiPeiFu Experimental Animal Co., Ltd. (Beijing, China). Animals were housed under controlled conditions (24 ± 2 °C, 70 ± 5% relative humidity, 12 h light/dark cycle) with free access to food and water. After a 7-day acclimation period, rats were randomly assigned to three groups (*n* = 5 per group) for IV, PO, or IP administration. All animals were fasted for 12 h prior to dosing, with free access to water. All experimental procedures were approved by the Animal Care and Use Committee of China Agricultural University and conducted in accordance with the Guide for the Care and Use of Laboratory Animals (National Research Council, Washington, DC, USA).

### 4.3. Preparation of Standard Solution and Quality Control Samples

Leonurine and the IS were separately dissolved in methanol to prepare stock solutions at 1 mg/mL, which were stored at 4 °C. The IS stock solution was further diluted with methanol–acetonitrile (1:1, *v*/*v*) to obtain a working solution at 5 ng/mL. Leonurine working solutions were prepared by serial dilution of the stock solution with methanol–acetonitrile (1:1, *v*/*v*). Calibration standards were prepared by spiking appropriate volumes of leonurine working solutions into blank biological matrices to yield final concentrations of 0.5, 1, 2, 5, 20, 50, 200, 500, and 1000 ng/mL. QC samples were prepared at 2, 20, and 400 ng/mL (low, medium, and high QC levels, respectively).

### 4.4. Drug Administration and Biological Samples Collection

#### 4.4.1. Dosing and Biological Sample Collection

For IV and IP administration, leonurine was dissolved in a vehicle consisting of 10% DMSO and 90% hydroxypropyl-*β*-cyclodextrin at 1.5 mg/mL. For PO administration, leonurine was suspended in 0.5% methylcellulose at 2 mg/mL. Doses were 1.5 mg/kg (IV), 20 mg/kg (PO), and 15 mg/kg (IP). Blood samples (0.5 mL) were collected from the suborbital venous plexus at 0.083 h (IV only), 0.25, 0.5, 1, 2, 4, 8, and 24 h post-dose. Plasma was obtained by centrifugation at 3500 rpm for 10 min. Urine and feces were collected over 0–4, 4–8, 8–24, 24–32, and 32–48 h intervals. Feces samples were homogenized with saline at a ratio of 1:4 (*w*/*v*) prior to analysis.

#### 4.4.2. Sample Preparation

For pharmacokinetic and excretion analysis, 20 μL plasma, 50 μL urine, or 50 μL fecal homogenate was mixed with 300 μL IS working solution, vortexed for 2 min, and centrifuged at 13,500 rpm for 15 min. A 70 μL aliquot of the supernatant was diluted with 140 μL water.

For metabolite profiling, biological samples from the same treatment group were pooled using the AUC-weighted pooling method [28,29]. Samples were purified using solid-phase extraction (SPE). Cartridges were conditioned with 2 mL methanol followed by 2 mL water, after which samples were loaded, washed sequentially with 3 mL water and 3 mL methanol. The methanol eluates were evaporated to dryness under nitrogen at room temperature, reconstituted in 100 μL of 5% acetonitrile in water, centrifuged, and the supernatants were subjected to analysis.

### 4.5. Instrument and Conditions

#### 4.5.1. Pharmacokinetics and Excretion Study

Chromatographic separation was performed on an Exion LC system (Sciex, Framingham, MA, USA) using a Waters XSelect HSS T3 column (2.1 × 50 mm, 2.5 μm) at 40 °C. The mobile phase consisted of 0.1% formic acid in water (solvent A) and 0.1% formic acid in acetonitrile (solvent B), delivered at a flow rate of 600 μL/min under the following gradient program: 0–0.3 min, 5% B; 0.3–2.5 min, 5–95% B; 2.5–4 min, 95% B; 4–4.1 min, 95–5% B; 4.1–5.0 min, 5% B. The injection volume was 1 μL. The autosampler was set at 4 °C.

Mass spectrometric detection was performed using an AB SCIEX Triple Quad 6500 plus mass spectrometer utilized in positive ion mode. Optimized ion source parameters were configured as follows: ion spray voltage, 5000 V; turbo heater temperature, 400 °C; nebulizing gas (Gas 1), 50 psi; heating gas (Gas 2), 50 psi; curtain gas, 40 psi; and collision gas, 12 psi. Quantification was performed *via* multiple reaction monitoring (MRM) of the following transitions: *m*/*z* 312.3 → 181.0 for leonurine and *m*/*z* 472.2 → 436.5 for IS. A dwell time of 50 ms was assigned to each transition to ensure sufficient signal-to-noise ratio for accurate quantification. Data acquisition and processing were conducted using Analyst Software (version 1.7.2).

#### 4.5.2. Metabolism Study

Liquid chromatography analysis was performed using an Ultimate 3000 UHPLC system (Thermo Fisher Scientific, Waltham, MA, USA) equipped with a binary pump, an autosampler, and a thermostated column compartment. Chromatographic separation was carried out on a Waters ACQUITY T3 column (2.1 × 100 mm, 2.5 μm; Waters Corporation, Milford, MA, USA) maintained at 40 °C. The mobile phase consisted of 0.1% formic acid in water (solvent A) and 0.1% formic acid in acetonitrile (solvent B), delivered at a flow rate of 400 μL/min under the following gradient program: 0–2 min, 5% B; 2–15 min, 5–30% B; 15–17 min, 30–95% B; 17–19 min, 95% B. The injection volume was 10 μL.

Mass spectrometric data were acquired using a Q-Exactive hybrid quadrupole-Orbitrap mass spectrometer (Thermo Fisher Scientific, San Jose, CA, USA) equipped with a heated electrospray ionization source operated in positive and negative ion mode, respectively. A data-dependent MS/MS acquisition method was employed with the top five most intense precursor ions selected from each full MS scan for fragmentation. Full MS scans were recorded at a resolution of 70,000 FWHM, and MS/MS spectra at 17,500 FWHM. High-purity nitrogen was used as sheath gas (30 arbitrary units), auxiliary gas (10 arbitrary units), and collision gas. A step-normalized collision energy of 10, 30, and 50% was applied. Additional source parameters were configured as follows: mass scan range, *m*/*z* 100–750; spray voltage, ±3.5 kV; capillary temperature, 350 °C; heater temperature, 350 °C; and S-lens RF level, 55 V.

### 4.6. Method Validation

Method validation was performed according to standard bioanalytical guidelines. Selectivity, linearity, accuracy, precision, extraction recovery, matrix effect, and stability were evaluated in rat biological matrices. Calibration curves were constructed using weighted (1/*x*^2^) linear regression. The LLOQ was defined as the lowest concentration quantified with accuracy and precision within ±15%. To assess the precision and accuracy of the developed method, QC samples at three concentration levels (2, 20, and 400 ng/mL) were analyzed in five replicates for intra-day and inter-day evaluations. Inter-day precision and accuracy were evaluated using QC samples at three concentration levels, with five replicates at each level, across three independent analytical batches. Accuracy was expressed as the relative error (%), while precision was reported as the relative standard deviation (%). Extraction recovery of leonurine was determined at each QC level by comparing the peak area ratios (analyte to IS) of pre-extraction samples with those of post-extraction spiked samples. Matrix effects were assessed by comparing the peak responses of post-extraction spiked samples (A) with those of neat standard solutions at equivalent concentrations (B), with results expressed as (A/B × 100%). Storage stability in rat plasma was evaluated under three critical conditions: room temperature (24 ± 2 °C) for 4 h, autosampler condition (4 ± 2 °C) for 48 h, and long-term storage for 20 days (−80 ± 20 °C).

### 4.7. Pharmacokinetic Analysis

Pharmacokinetic parameters were derived using Phoenix WinNonlin software (version 6.2; Pharsight Corporation, Mountain View, CA, USA) using a noncompartmental model. For the IV administration group, the following parameters were estimated: C_0_, AUC_0–∞_, T_1/2_, V_d_, MRT, and CL. For the PO administration group, the parameters included C_max_, T_max_, AUC_0–t_, AUC_0–∞_, T_1/2_, and absolute F.

## 5. Conclusions

This study provides an integrated assessment of the pharmacokinetics, excretion, and metabolic fate of leonurine in rats. Leonurine showed rapid systemic clearance, extremely low oral bioavailability, and only limited excretion as the unchanged parent drug, collectively indicating that extensive metabolism is the predominant determinant of its in vivo disposition. Metabolite profiling revealed a complex biotransformation network consisting of 30 leonurine-related components, including 12 glucuronidated metabolites, 12 sulfated metabolites, and several phase I products. Importantly, the results demonstrate that ester bond cleavage represents one of the major primary biotransformation pathways of leonurine, generating hydrolysis-derived intermediates that subsequently undergo further conjugative metabolism. Accordingly, leonurine biotransformation is not confined to direct glucuronidation and sulfation of the intact scaffold, but also involves extensive secondary metabolism of hydrolysis-derived fragments. By defining the major pathways governing leonurine disposition, this work advances current understanding of its in vivo behavior and provides a useful foundation for future studies on metabolic mechanisms, enzyme phenotyping, structural optimization, and the pharmacological or translational development of leonurine-related compounds.

## Figures and Tables

**Table 1:** Pharmacokinetic parameters of leonurine in rats.

Parameters	IV	PO	IP
Dosage (mg/kg)	1.5	20	15
T_max_ (h)	/	1.00 ± 0.87	0.33 ± 0.14
C_0_ (ng/mL)	1513 ± 428	/	/
C_max_ (ng/mL)	/	1.85 ± 1.45	1210 ± 263
AUC_0–t_ (h∗ng/mL)	170 ± 41.2	3.14 ± 1.82	1138 ± 130
AUC_inf_ (h∗ng/mL)	171 ± 41.0	N.C.	1141 ± 130
V_d_ (L/kg)	3.85 ± 0.76	/	/
T_1/2_ (h)	2.48 ± 0.37	N.C.	1.18 ± 0.13
MRT (h)	0.41 ± 0.12	/	0.89 ± 0.06
CL (mL/min/kg)	152 ± 38.8	/	/
F (%)	/	0.14 ± 0.08	66.6 ± 7.58

N.C.: not calculated; /: not applicable.

**Table 2:** Identification of leonurine metabolites in rats using UHPLC/Orbitrap MS.

Peak	t_R_ (min)	Ionization Mode	Experimental *m*/*z*	Error (ppm)	Formula [M-H]^−^ or [M+H]^+^	MS/MS Fragments	Biotransformation (Annotation)	IV			PO			IP		
								U	P	F	U	P	F	U	P	F
M1 ^#^	0.68	Pos	132.1134	−1.34	C_5_H_14_ON_3_	90.0919, 73.0654, 60.0562	Hydrolysis (4-guanidine-1-butanol)	+	+	+	+	+	+	+	+	+
M2 ^#^	2.12	Neg	373.0781	0.49	C_15_H_17_O_11_	197.0448, 182.0213, 175.0241, 166.9977	Hydrolysis, glucuronidation		+		+	+		+	+	
M3 *	2.14	Neg	197.0448	2.03	C_9_H_9_O_5_	182.0213, 166.9977, 123.0075	Hydrolysis (Syringic acid)								+	
M4 ^#^	2.14	Neg	277.0020	0.96	C_9_H_9_O_8_S	197.0448, 182.0213, 166.9978	Hydrolysis, sulfation		+					+	+	
M5 ^#^	2.57	Pos	474.1738	3.11	C_19_H_28_O_11_N_3_	298.1401, 167.0341, 132.1134, 114.1029	Demethylation, glucuronidation	+			+			+		
M6 ^#^	3.00	Pos	650.2053	2.24	C_25_H_36_O_17_N_3_	474.1730, 298.1402, 167.0341, 114.1029	Demethylation, diglucuronidation	+	+		+	+		+	+	
M7 ^#^	3.12	Pos	474.1722	−0.36	C_19_H_28_O_11_N_3_	298.1403, 167.0342, 132.1133, 114.1030	Demethylation, glucuronidation	+			+			+		
M8 ^#^	3.49	Pos	474.1726	1.23	C_19_H_28_O_11_N_3_	298.1402, 167.0341, 132.1134, 114.1030	Demethylation, glucuronidation	+	+		+	+		+	+	
M9 ^#^	3.64	Pos	474.1732	1.76	C_19_H_28_O_11_N_3_	298.1403, 167.0342, 132.1134, 114.1030	Demethylation, glucuronidation	+			+			+		
M10 ^#^	3.64	Pos	474.2098	1.21	C_19_H_28_O_11_N_3_	312.1560, 181.0499, 132.1134, 114.1030	Glucosylation							+		
M11	3.67	Neg	486.1738	0.58	C_20_H_28_O_11_N_3_	310.1403, 295.1176, 280.0931, 197.0448	Glucuronidation	+	+	+	+	+	+	+	+	
M12 ^#^	3.67	Neg	373.0771	0.17	C_15_H_17_O_11_	197.0447, 182.0211, 175.0240, 166.9976	Hydrolysis, glucuronidation							+	+	
M13 ^#^	3.87	Neg	500.1891	1.93	C_21_H_30_O_11_N_3_	324.1565, 309.1330, 197.0449	Methylation, glucuronidation	+			+			+	+	
M14 ^#^	4.03	Pos	298.1406	1.32	C_13_H_20_O_5_N_3_	167.0342, 132.1134, 114.1030, 97.0765	Demethylation	+		+	+		+	+		+
M15	4.18	Neg	390.0972	0.61	C_14_H_20_O_8_N_3_S	310.1404, 295.1167, 280.0933, 197.0448	Sulfation	+	+	+	+	+	+	+	+	
M16	4.34	Neg	486.1727	0.71	C_20_H_28_O_11_N_3_	310.1407, 295.1182, 280.0941, 197.0452	Glucuronidation	+	+		+	+		+	+	
M17 ^#^	4.36	Neg	324.1563	2.82	C_15_H_22_O_5_N_3_	309.1327, 294.1090, 197.0454	Methylation							+		
M18 ^#^	4.36	Neg	404.1128	−0.08	C_15_H_22_O_8_N_3_S	324.1561, 309.1327, 294.1093, 197.0449	Methylation, sulfation	+	+		+			+	+	
M19 ^#^	4.36	Neg	376.0815	0.37	C_13_H_18_O_8_N_3_S	296.1248, 281.1014, 183.0290	Demethylation, sulfation	+	+		+			+	+	
M20 ^#^	4.40	Neg	291.0179	3.21	C_10_H_11_O_8_S	211.0606, 196.0370, 181.0135	Hydrolysis, methylation, sulfation	+	+			+	+	+	+	
M21 ^#^	4.44	Neg	376.0816	0.56	C_13_H_18_O_8_N_3_S	296.1250, 281.1015, 183.0291	Demethylation, sulfation	+	+		+			+	+	
M22 ^#^	4.70	Neg	387.0930	4.70	C_16_H_19_O_11_	211.0605, 196.0369, 181.0133	Hydrolysis, methylation, glucuronidation		+		+	+		+	+	
M23 ^#^	4.77	Neg	277.0023	0.60	C_9_H_9_O_8_S	197.0449, 182.0213	Hydrolysis, sulfation		+						+	
M24 ^#^	4.93	Neg	404.1127	−0.15	C_15_H_22_O_8_N_3_S	322.1408, 206.0598, 197.0453, 80.9638	Methylation, sulfation	+	+		+			+	+	
M25 *	4.99	Neg	310.1407	1.07	C_14_H_20_O_5_N_3_	295.1172, 280.0938, 197.0448, 166.9979	Parent Drug	+	+	+	+	+	+	+	+	+
M26 ^#^	5.04	Neg	387.0932	1.19	C_16_H_19_O_11_	211.0602, 196.0370, 181.0133	Hydrolysis, methylation, glucuronidation		+						+	
M27 ^#^	5.16	Neg	307.0125	0.72	C_10_H_11_O_9_S	227.0550, 212.0315	Hydrolysis, methylation, oxidation, sulfation				+			+	+	
M28	5.36	Neg	390.0974	0.76	C_14_H_20_O_8_N_3_S	310.1405, 295.1171, 280.0935, 197.0448	Sulfation	+	+		+	+		+	+	
M29 ^#^	5.49	Neg	404.1129	0.47	C_15_H_22_O_8_N_3_S	324.1562, 309.1322, 294.1087, 197.0448	Methylation, sulfation	+			+			+		
M30 ^#^	5.52	Neg	291.0179	3.42	C_10_H_11_O_8_S	211.0605, 196.0370	Hydrolysis, methylation, sulfation							+	+	

*: Parent commercial obtained; ^#^: newly reported metabolites; +: detectable; U: urine; P: plasma; F: Feces.

## Data Availability

The original contributions presented in this study are included in the article/Supplementary Material. Further inquiries can be directed to the corresponding authors.

## Supplementary Materials

The following supporting information can be downloaded at: https://www.mdpi.com/article/10.3390/molecules31122002/s1, Table S1: Summary of accuracy, matrix effect and precision of leonurine in rat plasma. Table S2: Stability data for leonurine in rat plasma.
