# Supplementary material for: Pharmacokinetics, Excretion, and Metabolite Profiling of Leonurine in Rats: Evidence for Extensive Phase II Conjugations
Source: Molecules. 2026 Jun 8;31(12):2002. doi: 10.3390/molecules31122002 (PMC13305200; doi:10.3390/molecules31122002)
Supplement: Supplementary file 1 [file molecules-31-02002-s001.zip › molecules-4316741-supplementary.pdf]

Pharmacokinetics, Excretion, and Metabolite Profiling of Leonurine in Rats: Evidence for Extensive Phase II Conjugations

Xu Liu [1,†], Jing Hu [1,†], Yang Chen [2], Bin Shi [3], Zhanpeng Shang [4,*] and Yan Liang [1,*]

[1] Department of Pharmacy, Medical Supplies Center of PLA General Hospital, Beijing 100853, China; liuxu8222@163.com (X.L.); huj301@126.com (J.H.)
[2] Senior Department of Traditional Chinese Medicine, Chinese PLA Hospital, Beijing 100853, China; cydoctor@163.com
[3] Department of Organ Transplantation, The Third Medical Center of PLA General Hospital, Beijing 100039, China; hongb619@163.com
[4] School of Pharmaceutical Sciences, Peking University, Beijing 100083, China
* Correspondence: zpshang1206@bjmu.edu.cn (Z.S.); liang730924@163.com (Y.L.)
† These authors contributed equally to this work.

Table S1 Summary of accuracy, matrix effect and precision of leonurine in rat plasma

Spiked concentration (ng/mL)	Measured concentration (ng/mL)	Mean accuracy (%)	Mean matrix effect (%)	Intra-day precision, RSD (%)	Inter-day precision, RSD (%)	Mean recovery (%)	Repeatability (%)
2	1.94±0.084	97.3	93.1	7.03	4.25	98.0	3.94
20	17.4±0.252	87.1	95.6	4.25	4.17	88.1	5.53
400	368±2.08	92.1	101	4.20	3.28	89.9	2.65

Table S2 Stability data for leonurine in rat plasma

Storage condition	Spiked concentration (ng/mL)	Accuracy (%)
Room temperature (4 hr)	2	96.6
	20	93.6
	400	100.3
Autosampler for 24 hr (4°C)	2	89.7
	20	95.9
	400	101.2
Long-term stability (14 Day, -80°C)	2	92.2
	20	93.7
	400	99.9
